# The Emergence of Integrated Information, Complexity, and ‘Consciousness’ at Criticality

**DOI:** 10.3390/e22030339

**Published:** 2020-03-16

**Authors:** Nicholas J.M. Popiel, Sina Khajehabdollahi, Pubuditha M. Abeyasinghe, Francesco Riganello, Emily S. Nichols, Adrian M. Owen, Andrea Soddu

**Affiliations:** 1 Department of Physics and Astronomy, Western University, 151 Richmond St, London, ON N6A 3K7, Canada; sina.abdollahi@gmail.com (S.K.); enicho4@uwo.ca (E.S.N.); asoddu@uwo.ca (A.S.); 2 Faculty of Medicine Nursing and Health Sciences, Monash University, Wellington Rd, Clayton VIC 3800, Australia; pubu.abeyasinghe@monash.edu; 3 Research in Advanced Neurorehabilitation (RAN), S. Anna Institute, Via Siris 11, 88900 Crotone, Italy; francescoriganello@gmail.com; 4 Brain and Mind Institute, Western University, 151 Richmond St, London, ON N6A 3K7, Canada; aowen6@uwo.ca; 5 Department of Psychology and Department of Physiology and Pharmacology, 151 Richmond St, London, ON N6A 3K7, Canada

**Keywords:** Ising model, integrated information, criticality

## Abstract

Integrated Information Theory (IIT) posits that integrated information (Φ) represents the quantity of a conscious experience. Here, the generalized Ising model was used to calculate Φ as a function of temperature in toy models of fully connected neural networks. A Monte–Carlo simulation was run on 159 normalized, random, positively weighted networks analogous to small five-node excitatory neural network motifs. Integrated information generated by this sample of small Ising models was measured across model parameter spaces. It was observed that integrated information, as an order parameter, underwent a phase transition at the critical point in the model. This critical point was demarcated by the peak of the generalized susceptibility (or variance in configuration due to temperature) of integrated information. At this critical point, integrated information was maximally receptive and responsive to perturbations of its own states. The results of this study provide evidence that Φ can capture integrated information in an empirical dataset, and display critical behavior acting as an order parameter from the generalized Ising model.

## 1. Introduction

A growing body of evidence has emerged suggesting that many disparate natural, and particularly biological, phenomena reside in a critical regime of dynamics on the cusp between order and disorder [1,2,3,4,5,6,7,8,9,10,11]. More specifically, it has been shown that models tuned to criticality exhibit similar dynamics to the brain [12,13,14,15], which, has led to the emergence of the Critical Brain Hypothesis [8,10]. Systems tuned to criticality exhibit a number of useful informational properties that allow for the efficient distribution of, and susceptibility to, information [6,10,15,16,17]. For example, Marinazzo et al. demonstrated that information is maximized at the critical temperature in an Ising scheme using human connectome data and beyond criticality, a law of marginal diminishing returns is reached [15]. These ideas have been further developed to suggest more broadly that critical systems are evolutionarily advantageous in that they are more effective at reacting to their environment and ensuring their continued survival [18,19,20].

Integrated information theory (IIT) is a top-down, phenomenological approach to defining consciousness [21]. Starting from phenomenological axioms inherently true for all conscious experiences, mathematical postulates have been put forth creating a workspace for quantitative research on consciousness. The main measure proposed by IIT 3.0 is the mathematical entity called integrated conceptual information (Φ) (Big Phi) which generally seeks to measure ‘how much the whole is greater than the sum of its parts’ of the causal structure being studied [21]. Though other measures exist [22] which attempt to capture some form of integration or complexity, this paper uses Φ as the metric quantifying conscious experience. For a comprehensive overview of the mathematical taxonomy of the possible variations in defining integrated information, see [23,24].

Integrated information is a type of complexity measure that quantifies how mechanisms in a system interact and constrain each other in emergent, irreducible ways. This allows us to measure the properties of a system that cannot be explained by independent components of that system. Φ is sensitive to not just information, which in general can be maximized by deterministic systems with unique pasts and futures, but also to the distribution and integration of information. In general, this tendency to distribute and integrate information is maximized by strongly coupled systems. To have a system that is both strongly coupled and informative requires a balance between segregating forces acting to differentiate the system, in conjunction with integrating forces creating new forms of information not present within the individual components. In the Ising model, it is expected that these exact properties emerge near the critical temperature at the onset of a phase transition.

It is important to note here that extending the concept of criticality to IIT is not new. Aguilera has studied criticality and the behavior of Φ in a classical Ising model with a constant temperature [25]. Furthermore, Kim and Lee have noted that criticality and Φ are correlated in a Kuramoto model along with EEG [26]. In their study coupling strength was used as the order parameter to achieve criticality whereas here, a random set of connectivity matrices were used, and the temperature was varied as the critical parameter. Because temperature was used as the critical parameter, we could test the critical behavior of Φ independent of network connectivity.

The generalized Ising model simulates brain dynamics through its ability to exhibit phase transitions and critical points and is the simplest model associated with empirical pairwise correlation data [18,27]. Historically, the 2D classical Ising model exhibits a phase transition at a critical temperature $T_{c}$, a global scaling parameter of the model. This model, originally intended to describe the ferromagnetic properties of materials, was soon extended to different systems with binary interactions. Recently, it has been shown to exhibit similar dynamics to that of the brain, giving rise to the Critical Brain Hypothesis [2,3,4,5,6,7,8,9,10,12,14,16,28,29,30,31]. Fraiman et al. have shown that the concept of criticality extends to classical Ising motifs with N=200 nodes while modelling the brain [12].

This paper aims to demonstrate the critical properties of Φ extend to different network connectivities with dynamics governed by the generalized Ising model. The generalized Ising model has been shown to simulate the statistical behavior of the brain [12,13,14,15]. By generating Φ for random networks, a methodology is outlined that can be applied with patient tractographies to create a novel workspace for IIT.

To this end, the Ising model was simulated and criticality was obtained on 159 randomly generated, positive weighted *N* = 5 nodes networks to explore the combinatoric space of these neural network motifs. Each unique network displayed its own idiosyncratic phase transition as measured across a variety of its thermodynamic variables. Φ and its susceptibility $\chi_{\Phi }$ were measured across the parameter space of these models, fitting in naturally among the other thermodynamic variables such as energy (*E*), magnetization (*M*), specific heat ($C_{v}$) or magnetic susceptibility (χ). Simulations swept across the model’s only free parameter, the temperature of the surrounding heat bath. As the temperature was varied from low to high, increasing energetic fluctuations became more probable. In many cases, as this parameter was varied, the organizational structure of the system dramatically changed, exhibiting a phase transition. Phase transitions or generalizations of such are at the heart of many of the most interesting complex systems such as genetic networks [32] societal organizations, financial markets [33,34], or swarming behaviors [35,36]. The critical points where these transitions are located are demarcated by the ‘critical temperature’ ($T_{c}$) of the generalized Ising model.

In Equation (1) an overview of the strategy employed in this paper is summarized. Ising simulations were run given a connectivity matrix and temperature ($J_{ij}$, *T*) with the outputs: magnetization, magnetic susceptibility, energy, specific heat, the current spin configuration of the system, and, a Markovian transition probability matrix (TPM). These parameters were then used to calculate the criticality of the system along with integrated information and the susceptibility of integrated information. All parameters were calculated as a function of temperature [*M*, χ, *E*, $C_{v}$, TPM, s, Φ, $\chi_{\Phi }$](*T*).

(1) $$\begin{array}{cc} f_{\mathrm{Ising}}\left(J_{ij},T\right) & \to \left[M,\chi ,E,C_{v},\mathrm{TPM},\vec{s}\right]\left(T\right)\\ f_{\Phi }\left(\mathrm{TPM},\vec{s}\right)\left(T\right) & \to \left[\Phi ,\chi_{\Phi }\right]\left(T\right). \end{array}$$

## 2. Materials and Methods

### 2.1. Random Networks

159 fully connected networks of five nodes with random edge weights uniformly sampled between 0 and 1 were generated and the principal diagonal was set to 0. The networks were then normalized such that their strongest weight was always unity. These random networks were saved as connectivity matrices ($J_{ij}$) and fed into Monte–Carlo Metropolis Ising simulations. These random networks were designed to explore the combinatoric space of ’ferromagnetic’ (positively weighted $J_{ij}$) fully connected neural network motifs each of which was constrained to 5 nodes.

This class of network was chosen as they are computationally tractable and represent brain connectivity as obtained through tractography measures. The calculation of integrated information scales super-exponentially in the number of nodes growing on $O(n53^{n})$ [37]. By choosing networks of 5 nodes, the computational intractability was overcome. Following the methodology of Marinazzo et al. and Abeyasinghe et al., the $J_{ij}$ can be obtained through tractography measures by counting the number of fibers connecting the $i^{th}$ and $j^{th}$ regions [15,31]. This results in the primary diagonal always being 0 (since one region has no fibers connecting it to itself). We further restrict ourselves to ferromagnetic connectivity matrices which correspond to the assumption of having only excitatory interactions. Although most parcellations of the brain are larger than 5 × 5, this treatment can be extended to network-level dynamics.

### 2.2. Ising Model

The Ising model is a simple way to simulate many-body interactions between binary elements. The simplified Ising model takes the form of Equation (2), where it is important to note in the classical model, the (i,j) indices correspond to only neighboring lattice sites and in the general model, this restriction is relaxed. The equations do not change from model to model, instead, it is only a change in the collective interaction between lattice sites.

(2) $$\begin{array}{c} H=-\sum_{i,j}s_{i}s_{j}J_{ij}. \end{array}$$

In this equation, $J_{ij}$ corresponds to the weights of nodal connections, $s_{i}=\pm 1$ representing the binary state of a magnetic ’spin’ site, and, *H* is the Hamiltonian or microscopic (dependent on spin configuration) energy.

In the most generic form, the Ising model also contains a magnetic field term. This term was not included in this study as we followed the protocol of Fraiman et al. and Abeyasinghe et al. for simulating spontaneous brain activity [12,31]. Present studies in our Lab are investigating the introduction of an external magnetic field to simulate an external stimulation.

When applying the Ising model to the brain, $J_{ij}$ is the number of fibers as determined through tractography measures for each region of interest, and each region of interest’s dynamics are represented as the spin state $s_{i}$.

Equation (2) was used alongside nature’s tendency to minimize energy to form the transition probability matrices for each of the 159 random networks. To equilibrate the model, a Monte–Carlo Metropolis algorithm and corresponding update rules were applied [38]. Through each iteration, a random element in the model was chosen and allowed the possibility for a ‘spin-flip’. A spin-flip will occur if the energy of the system after the flip is favorable (decreases). If the energy increases, the Boltzmann factor ($k_{B}=1$ in this study) was used to assign the probability that the ’spin-flip’ occurs.

By using the Monte–Carlo Metropolis algorithm, the calculation of the system’s partition function was not needed, as only a ratio of probabilities was required. Equation (3) describes the probability that was assigned to the *i*-th ’spin-flip’ location.

(3) $$\begin{array}{c} \begin{array}{ccc} \mathrm{Prob}\left(s_{i}\to -s_{i}\right) & =\exp\left(-\frac{\Delta E}{k_{B}T}\right) & \mathrm{if}\;\Delta E>0\\ & =1 & \mathrm{if}\;\Delta E\leq 0. \end{array} \end{array}$$

Following the update rules of Equation (3), a ’state-by-node’ TPM was constructed. The temperature in the model affects the rate at which ‘unfavorable’ spin-flips occur; increasing the temperature increases the noise/randomness of the model’s dynamics. Within each time step, all spins could flip once, updating simultaneously for the next step until the process was repeated for the desired number of time steps. Once the system had had enough time to equilibrate past its transient initial state, observables in the model were measured repeatedly and accumulated to generate the equilibrium expectation values.

Throughout the study, the simulation was run for an initial 500 time points to allow for thermalization. Upon equilibrating, the simulation was run for 2000 iterations. Thermodynamic properties and transition probabilities were calculated on each iteration. A logarithmically scaled temperature distribution was sampled 200 times between 0.1 and 4, with the simulation rerun for each temperature.

### 2.3. Summary Statistics

The summary statistics for observables O measured in this experiment are defined below. The thermodynamic observables (*M*,χ) are stated for completeness.

(4) $$\begin{array}{c} \begin{array}{cc} M= & \frac{1}{N}\left|\sum_{i=1}^{N}s_{i}\right| \end{array} \end{array}$$

(5) $$\begin{array}{c} \begin{array}{cc} \chi = & \frac{\langle M^{2}\rangle -{\langle M\rangle }^{2}}{T} \end{array} \end{array}$$

(6) $$\begin{array}{c} \begin{array}{cc} \langle O_{t}\rangle = & \frac{1}{N_{t}}\sum_{i}^{N_{t}}O_{i} \end{array} \end{array}$$

(7) $$\begin{array}{c} \begin{array}{cc} \chi_{O}= & \langle O^{2}\rangle -{\langle O\rangle }^{2}=\sigma_{t}^{2}\left(O\right) \end{array} \end{array}$$

(8) $$\begin{array}{c} \begin{array}{cc} \sigma_{J}^{2}\left(O\right)= & {\langle {\langle O\rangle }^{2}\rangle }_{J}-{\langle \langle O\rangle \rangle }_{J}^{2}, \end{array} \end{array}$$

where $\langle O_{t}\rangle$ is the expectation value of an observable across each iteration t. $\chi_{O}$ is the generalized susceptibility [19,39,40] and $\sigma_{J}^{2}\left(O\right)$ is the variance of an observable across all networks. All these summary statistics are calculated throughout the simulation.

### 2.4. Phi

Integrated Information (Φ) was calculated (using the PyPhi Python toolbox [37]) in the 5-node Ising model for 2000 iterations after the model reached a steady-state, which was assumed to be achieved after 500 iterations. To calculate Φ a TPM must be supplied as well as the configuration of the system at that time-step ($s_{t}$). Calculating the TPM required the calculation of probabilities from any configuration to any other by iterating Equation (3) across all spin sites that needed to flip for the transition to occur, then taking their product. This led to a state-by-node TPM, which was the input parameter for PyPhi. These transition probabilities specified and constrained the past, present, and, future states of the system. In the framework of IIT 3.0 and PyPhi, ‘the TPM completely specifies the system’ [37], once the TPM has been realized, Φ is readily determined.

Integrated information is defined through the effective information (ϕ) of the minimum information partition (MIP). This is the partition made to the system that results in the minimum effective information. Φ is then a function of the total system *X*, the state of the system *x* and the partition *P*. The basic schematic of calculating Φ is outlined below and more detail is available in [21,23,24,25,26].

(9) $$\begin{array}{c} \begin{array}{cc} \Phi \{X;x\}= & \phi \{X;x,\mathrm{MIP}(x)\} \end{array} \end{array}$$

(10) $$\begin{array}{c} \begin{array}{cc} \mathrm{MIP}(x)= & \min\left[\phi (X;x,P)\right]. \end{array} \end{array}$$

## 3. Results

Results indicated that the integrated conceptual information generated using the generalized Ising model, much like the classical variable magnetization, underwent a phase transition at the critical temperature. This was detected by locating the peaks of its susceptibility curves as a function of temperature [41], indicating that the integrated information structure of simple neural networks behaves critically, exhibiting maximal susceptibility to perturbations and allowing for a form of consciousness that balances coherence and continuity with information and variance. These results fit into the scheme in which the nature of evolution and the adaptive advantage of critical systems are understood in the context of a universe undergoing a cascade of phase transitions [42,43]. In statistical mechanics, phase transitions are typically thought to occur in the large *N* limit. However, Fraiman et al. have shown that the Ising model behaves critically for *N* = 200 nodes. In the attached supplementary material (Appendix A), it is demonstrated that when simulating the generalized Ising model for *N* = 525,100,250 nodes the same critical behavior is displayed [12].

159 Ising simulations were run on fully connected randomly weighted networks with *N* = 5 nodes. Summary statistics were calculated for simulated variables as a function of the fitting parameter *T*: magnetization *M*, integrated information Φ, the magnetic susceptibility χ, the generalized susceptibility of integrated information $\chi_{\Phi }$, along with the variances across all random network samples $\sigma_{J}^{2}\left(O\right)$.

**Figure 1:**
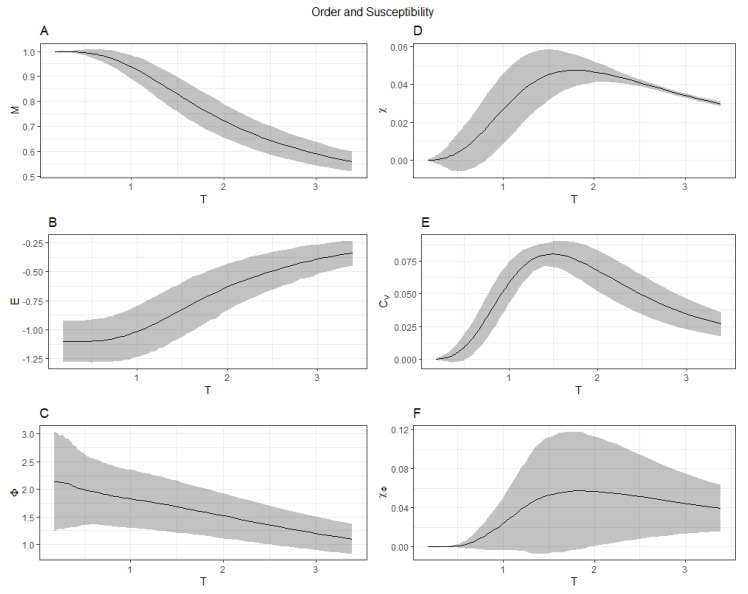
The summary statistics for the three order parameters, Magnetization *M*, energy *E* and Φ (**panels A**–**C**) across all the 159 random network simulations are shown. The variance of Φ, $\chi_{\Phi }=\sigma_{t}^{2}\left(\Phi \right)$ (**panel F**) is interpreted as a susceptibility of Φ and is compared to the magnetic susceptibility χ (**panel D**). Another critical parameter, the specific heat $C_{v}$ is plotted in **panel E**. These susceptibilities peak at the same critical temperature $T_{c}=1.8$ indicating the phase transition of integrated information as an order parameter in the Ising model. Error bars represent standard deviation of parameters across each connectivity matrix.

Figure 1 displays the summary statistics for the order and susceptibility parameters of the random networks. Magnetization *M*, energy *E*, and Φ are plotted in the left-most column. Susceptibility χ, specific heat $C_{v}$, and the susceptibility of Φ, $\chi_{\Phi }$ are plotted in the right-most column. By averaging these variables across all random networks, a strong parallel between the behavior of magnetization *M* and integrated information Φ can be seen. Near the onset of criticality (generally approximated by the peak of the magnetic susceptibility curve [40]) integrated information, much like the magnetization in the Ising model, undergoes a phase transition denoted by the peak in the susceptibility of Φ ($\chi_{\Phi }$). The regime where the fluctuations of integrated information are maximized suggests a transition point for integrated information as an order parameter, demonstrating that the phenomenon of criticality extends into the behavior of integrated information in the Ising model.

**Figure 2:**
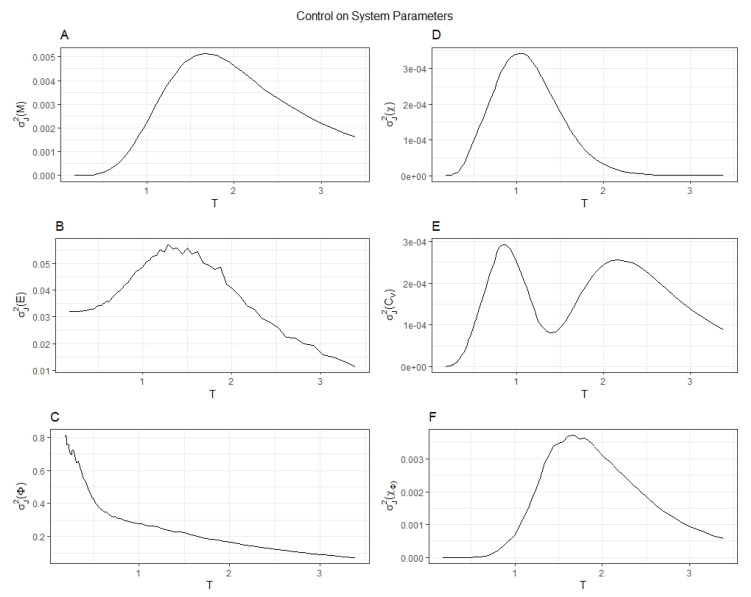
The variance of the order parameters *M*, *E*, and Φ (**panels A**–**C**) and their susceptibilities (**panels D**–**F**) across different connectivities are plotted. These plots demonstrate the potential control one can impart to the Ising model by changing the connectivity matrix.

In Figure 2 the variance of magnetization ($\sigma_{J}^{2}\left(M\right)$), energy ($\sigma_{J}^{2}\left(E\right)$), and integrated information ($\sigma_{J}^{2}\left(\Phi \right)$) are plotted in the left-most column. The variance of magnetic susceptibility ($\sigma_{J}^{2}\left(\chi \right)$), specific heat ($\sigma_{J}^{2}\left(C_{v}\right)$), and the susceptibility of integrated information ($\sigma_{J}^{2}\left(\chi_{\Phi }\right)$) are plotted in the right-most column. All variances were taken across the different random network connectivities. The variances $\sigma_{J}^{2}$ illustrate how the connectivity choice affected the order parameters across random networks, whereas the susceptibilities quantify the mean fluctuations of each order parameter averaged across all random networks. These summary statistics give insights into the behavior of simple fully connected neural networks. $\sigma_{J}^{2}\left(\Phi \right)$ shows to distinct regions, the first at low temperatures corresponds to a steep fall off and the second at higher temperatures is a linear decrease. These results highlight the regions where changes in the structural connectivity of the model had the most influence on the generation of integrated information. At lower temperatures, there is a much greater effect on Φ from different connectivities than at higher temperatures. On the other hand, $\sigma_{J}^{2}\left(\chi_{\Phi }\right)$ displays the same trend as $\sigma_{J}^{2}\left(\chi \right)$ where near the critical temperature there is an increase in variance suggesting multiple network connectivities exhibit this critical behavior. As the networks became increasingly irreducible at criticality, each component contributed more to the conscious experience. While the magnetization of the model near criticality was maximally sensitive to changes in the structural connectivity, whereas integrated information instead decays linearly.

## 4. Discussion

### 4.1. Phase Transitions IIT

To investigate the properties of integrated information formulated by IIT 3.0, the Ising model was employed to act as a proxy to the brain. The results show that integrated information, as with magnetization tends to be maximally susceptible at the critical temperature (Figure 1). The ‘susceptibility’ of Φ(T), ($\chi_{\Phi }\left(T\right)$) has a distinct peak at criticality, typically the marker for a second-order phase transition with the classical 2D Ising model. This indicates that integrated information can be considered a macroscopic order parameter that undergoes a phase transition at the critical temperature. The present study was limited to implementing the Ising model on fully connected graphs using the Monte–Carlo Metropolis algorithm. In the future as more efficient algorithms for calculating Φ emerge (or as a compromise accurate correlates of Φ), combined with Monte–Carlo and network renormalization group methods [16,44,45,46,47,48,49], the exploration of larger networks of different classes (e.g., sparse, modular hierarchical, small-world, fractal) could lead to the identification of a rich taxonomy of phases of integrated information.

### 4.2. Evolution Complexity

The exploration of integrated information in the context of critical systems undergoing phase transitions motivates several new questions regarding the relationship between evolution, complexity, and consciousness. In the work done by Joshi et al. and Albantakis et al. on the complexity and the evolution of neural models and integrated information, it was shown that fitness can correlate strongly with Φ when the system is constrained in size and/or resources [50,51]. While it is not always true that a system will evolve to generate high Φ under more liberal constraints (e.g., infinite resources), it does seem to be that there may be some evolutionary advantage for having high Φ. Because Φ essentially measures the emergence of higher-order concepts within a system, it is not surprising that systems capable of generating higher-order concepts are also capable of representing and reacting to a diverse set of states. Therefore, for resource-limited systems, having an efficient means to represent internal and external states may automatically give rise to high Φ or ‘consciousness’.

### 4.3. Utility of Criticality

Critical systems have diverging correlation lengths, undergo critical slowing down (i.e., integration in space and time), and simultaneously exhibit distinct and segregated structures at all scales (i.e., scale-invariance). They are generally found in regimes of systems undergoing a transition between phases (e.g., magnetized vs. non-magnetized in the Ising model, or synchrony vs. asynchrony in the Kuramoto model [52,53,54,55,56]). In contrast to sub-critical regimes which can become completely uniform due to their strong coupling (high integration, low differentiation), and super-critical regimes which can become completely noise-driven (low integration, high differentiation), critical systems sit at the cusp of integration and differentiation; generating non-negligible Φ that is maximally susceptible to the perturbations of its environment and its own state. Our results indicate that while sub-critical regimes can generate high Φ, the variations in Φ are negligible. Only near the critical point does Φ have both large values and large fluctuations indicating that the critical point of the system is maximally receptive and responsive to its states. It is in this state of maximal susceptibility where concepts become maximally irreducible—$\chi_{\Phi }\left(T\right)$ increases to a peak. At this point of criticality, the ‘conscious experience’ as defined by IIT 3.0 is the most ’conscious’; criticality exhibited by the neural network motif leads to the ‘best’ conscious experience [21].

### 4.4. Future Work

Both the magnitude and susceptibility of Φ in the Ising model (and in general) are extremely sensitive to the connectivity of the underlying system. So far, simulations have been run on static networks, but in general, one can allow the network to evolve. Future work regarding how the networks arise could explore different evolutionary algorithms and dynamical rules in combination with the analysis from IIT. This would assess the role of evolution and the environment in generating Φ and its underlying critical structure. Exploring the behavior of Φ in different classes of phase transitions would allow further development of the ideas behind the Critical Brain Hypothesis, and would combine the fields of neuroscience, complexity science, material science, and statistical mechanics in order to understand the brain. Furthermore, the methodology of extracting Φ from the generalized Ising model can be applied using $J_{ij}$’s extracted from human cortical data, allowing Φ to be extracted for conscious individuals.

## 5. Conclusions

Ultimately, this study is best framed in the context of the emerging complexity of our world [43]. The brain is one of the most complex objects ever studied and the theory of it acting critically is gaining credence. New research into critical systems has shown that criticality may be useful for learning [9], and for optimizing information processing [10,18,19], both of which are tasks the brain is constantly performing. Phase transitions and criticality are gaining more relevance, and the evidence in this paper demonstrates that by defining consciousness with IIT and using the Ising model as a substrate, ‘consciousness’ undergoes a phase transition at criticality in the investigated neural network motifs. This, when combined with evidence that the brain may be critical, suggests that ‘consciousness’ may simply arise out of the tendency of the brain to self-organize towards criticality. The Critical Brain Hypothesis and IIT appear to go hand-in-hand, paving the way for more studies concerning the interdependencies between the two models in both patient and simulated data.

## Figures and Tables

**Figure A1:**
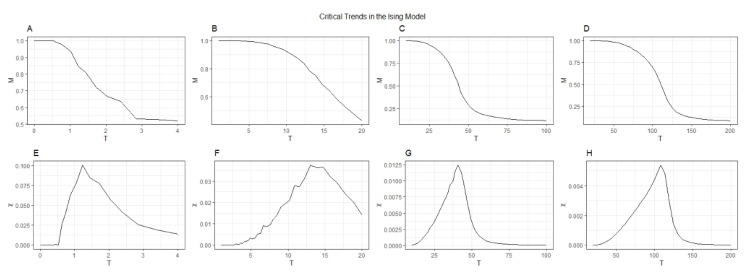
The magnetization *M*, for networks with *N* = 5 (**A**), 25 (**B**), 100 (**C**) and 250 (**D**) for random ferromagnetic networks. The magnetic susceptibility χ for each network is shown in **panels E**–**H**. Each network has a different critical temperature, but the same behaviour in susceptibility emerges independent of nodal size.

## Appendix A

The generalized Ising model outlined in this paper was deemed to be reach criticallity. However, the phenomenon of criticality is normally only present in the large *N* limit—is 5 nodes sufficient to make critical claims?

The following figures demonstrate that indeed, due to the generalization of the Ising model, critical claims are still valid. Recalling the seminal paper by Fraiman et al. regarding the classical Ising model and its relation to brain dynamics, criticality was claimed to be observed when simulating an Ising model on a square lattice with *N* = 200 nodes [12]. In the following plots, the peak in magnetic susceptibility χ is demonstrated at critical temperatures for the generalized Ising model with nodal sizes *N* = 525,100,250. For each network, the simulation was run for 50 temperatures. The parameters used in the simulation are summarized in Table A1.

**Table A1:** Summary of temperature parameters used in A1. 50 logarithmically scaled samples were used between $T_{i}$ and $T_{f}$.

N	$T_{i}$	$T_{f}$
5	0.001	4
25	1	20
100	10	100
250	20	200

As can be seen in Figure A1, a peak in susceptibility is present in all figures, indicating criticality is achieved up to the literature-accepted 200 node network. This demonstrates that the concept of criticality in the generalized Ising model extends beyond the classical large *N* limit, and the claims made in the manuscript regarding the apparent critical behavior of Φ are valid.
